# Small intestinal metastasis from primary breast cancer: a case report and review of literature

**DOI:** 10.3389/fimmu.2024.1475018

**Published:** 2024-12-03

**Authors:** Fengqing Shen, Songxiang Wang, Shanlu Yu, Yuancong Jiang

**Affiliations:** ^1^ Department of Breast and Thyroid Surgery, Shaoxing People’s Hospital, Shaoxing, China; ^2^ Department of Pathology, Shaoxing People’s Hospital, Shaoxing, China

**Keywords:** pleomorphic lobular carcinoma, small intestinal metastasis, chemotherapy, surgery, HER2 low expression, trastuzumab deruxtecan

## Abstract

Small intestinal metastasis from primary breast cancer remains a rare clinical occurrence. Despite extensive research into its clinicopathological features and treatment options, the specific pathogenesis and optimal management strategies remain incompletely understood. This case report presents a patient with breast cancer that metastasized to the small intestine. The primary breast tumor was diagnosed as classic invasive lobular carcinoma. Subsequent surgical intervention successfully addressed the intestinal obstruction and confirmed the metastatic origin of the small intestinal tumor. Interestingly, the metastatic lesions exhibited features suggestive of pleomorphic lobular carcinoma. A PET-CT scan was performed to evaluate the distant metastasis status of this patient. Notably, hormonal receptor status shifted from positive to negative, while HER2 expression changed from negative to low between the primary tumor and metastatic lesions. The presence of an undiagnosed pleomorphic component in the primary tumor might explain the disease’s progressive nature. In this case, systemic treatment with trastuzumab deruxtecan yielded favorable therapeutic outcomes. Overall, our findings suggest that re-evaluation of receptor status in breast cancer metastases is crucial for tailoring treatment strategies. Furthermore, a combination of palliative resection of small intestinal metastases and targeted therapy for HER2-low breast cancer may potentially improve survival.

## Introduction

Breast cancer remains the leading cause of cancer-related morbidity and mortality among women globally (1). While the brain, bones, lungs, and liver are well-recognized sites of metastatic spread (2), small intestinal metastasis from breast cancer is an exceptionally rare occurrence. Consequently, treatment strategies for this condition remain uncertain (3), with limited case reports available in the literature. Autopsy studies have revealed gastrointestinal tract involvement in approximately 6% to 18% of breast cancer metastasis cases, often associated with a poor prognosis (4, 5). Clinically, patients with small intestinal breast cancer metastases frequently present with symptoms such as intestinal obstruction or upper abdominal pain, which can be challenging to differentiate from primary intestinal tumors (4, 6). Small bowel resection is commonly recommended to alleviate symptoms. This case report describes a patient who was admitted to our hospital with upper abdominal discomfort and subsequently diagnosed with metastatic pleomorphic lobular carcinoma from the breast in the small intestine.

## Case presentation

**Figure 1:**
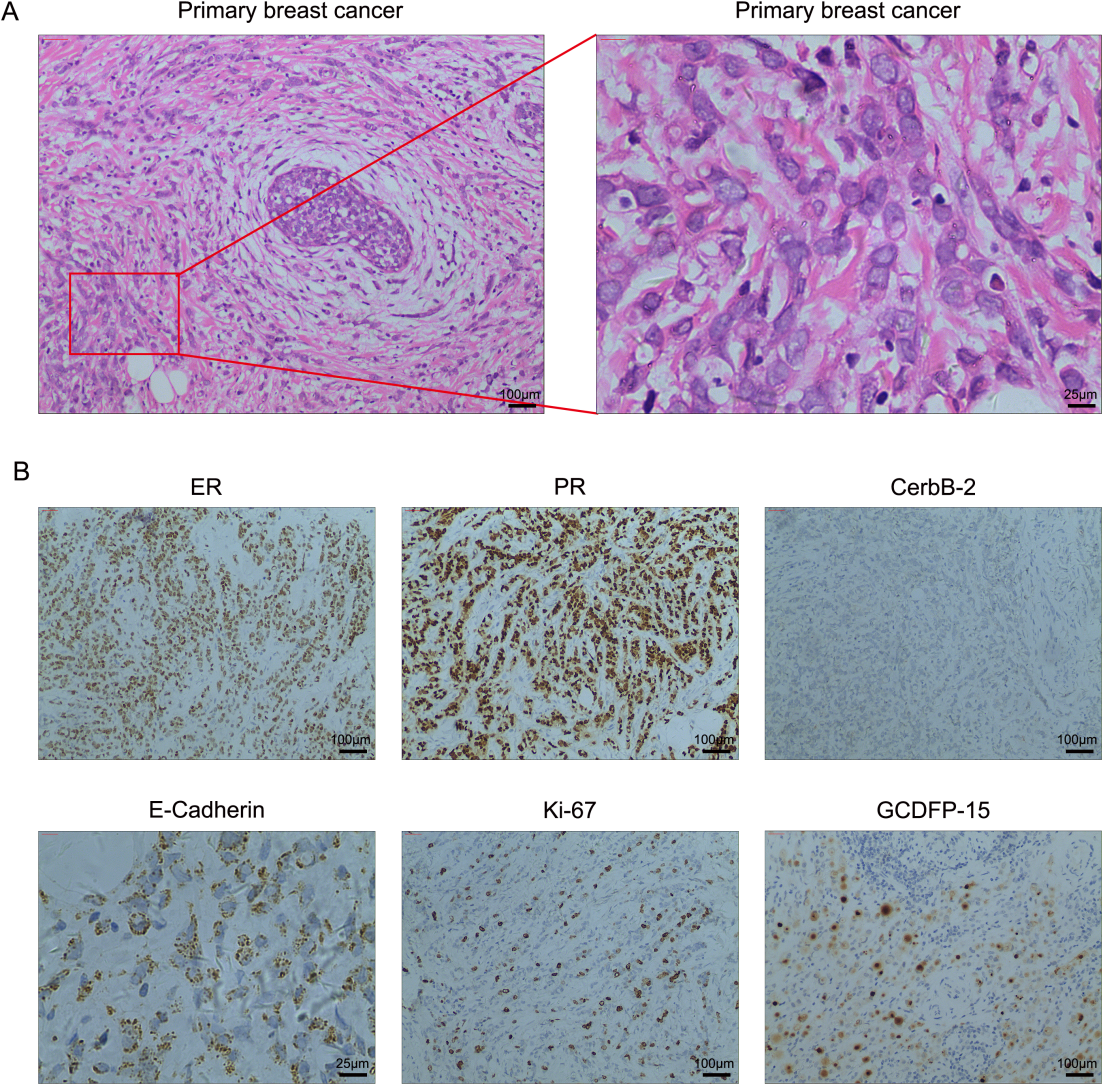
**(A)** High-resolution HE images of the primary breast cancer. **(B)** Representative immunohistochemical staining in breast cancer tissues. Magnification, 100×; scale bar, 100μm and magnification, 400×; scale bar, 25μm.

A 50-year-old woman presented to the hospital in December 2018 with a painless left breast mass. A left-modified radical mastectomy was performed, followed by an eight-day hospitalization. The postoperative pathology revealed classic invasive lobular breast carcinoma with the following immunohistochemical profile: ER (90% ++-+++), PR (95% +++), Ki-67 (+30%), CerbB-2 (-), E-Cadherin (+), and GCDFP-15 (+). Additionally, left axillary lymph node involvement was noted (27/28). Representative hematoxylin and eosin (HE) and immunohistochemical images are depicted in **Figure 1**. Subsequently, the patient underwent four cycles of adjuvant chemotherapy consisting of Epirubicin 90mg/m^2^ and Cyclophosphamide 600mg/m^2^, with an interval of 21 days between each cycle. Then, four additional cycles of Paclitaxel 175mg/m^2^ were used to enhance treatment efficacy. Due to abnormal liver function, the Paclitaxel dosage was adjusted to 160mg/m^2^. Subsequently, the patient received endocrine therapy with Anastrozole 1mg once a day and Goserelin 3.6mg once every 28 days, along with local radiotherapy.

In June 2023, the patient presented with upper abdominal discomfort. Physical examination revealed a palpable mass in the right abdomen. Laboratory results indicated elevated carcinoembryonic antigen (CEA), carbohydrate antigen 199 (CA-199), and carbohydrate antigen 125 (CA-125) levels at 5.24ng/ml, 58U/ml, and 48.2U/ml, respectively. A colonoscopy revealed congestion and edema of the terminal ileum mucosa, resulting in luminal narrowing. Subsequent CT imaging revealed diffuse thickening of the intestinal wall in the transverse colon, ascending colon, and terminal ileum, accompanied by blurred surrounding fat spaces, patchy shadows, and multiple enlarged mesenteric lymph nodes. The intestinal lumen remained patent. The pelvic viscera appeared normal, with no fluid accumulation in the abdominal cavity. However, a small amount of fluid was present in the pelvis, along with multiple small lymph nodes adjacent to the abdominal aorta. No overt lymphadenopathy was observed in the retroperitoneum or pelvic wall. Osteolytic bone destruction of the 12th thoracic vertebra, bilateral iliac bones, and sacrum suggested the possibility of metastatic tumors. To evaluate the distant metastasis status of this patient, a PET-CT scan was performed. The PET-CT results demonstrated increased glucose metabolism in the thickened intestinal wall of the lower ascending colon, consistent with a malignant lesion. Additionally, multiple strip-like densities and increased shadows at the mesenteric root, along with elevated glucose metabolism, suggested the possibility of peritoneal metastasis. Furthermore, widespread bone destruction with increased glucose metabolism and the formation of local soft tissue masses indicated multiple bone metastases. Based on these findings, an abdominal exploration was performed to confirm the diagnosis of small intestinal metastasis from breast cancer. The imaging data from CT, colonoscopy, and PET-CT are provided in **Supplementary Materials**.

**Figure 2:**
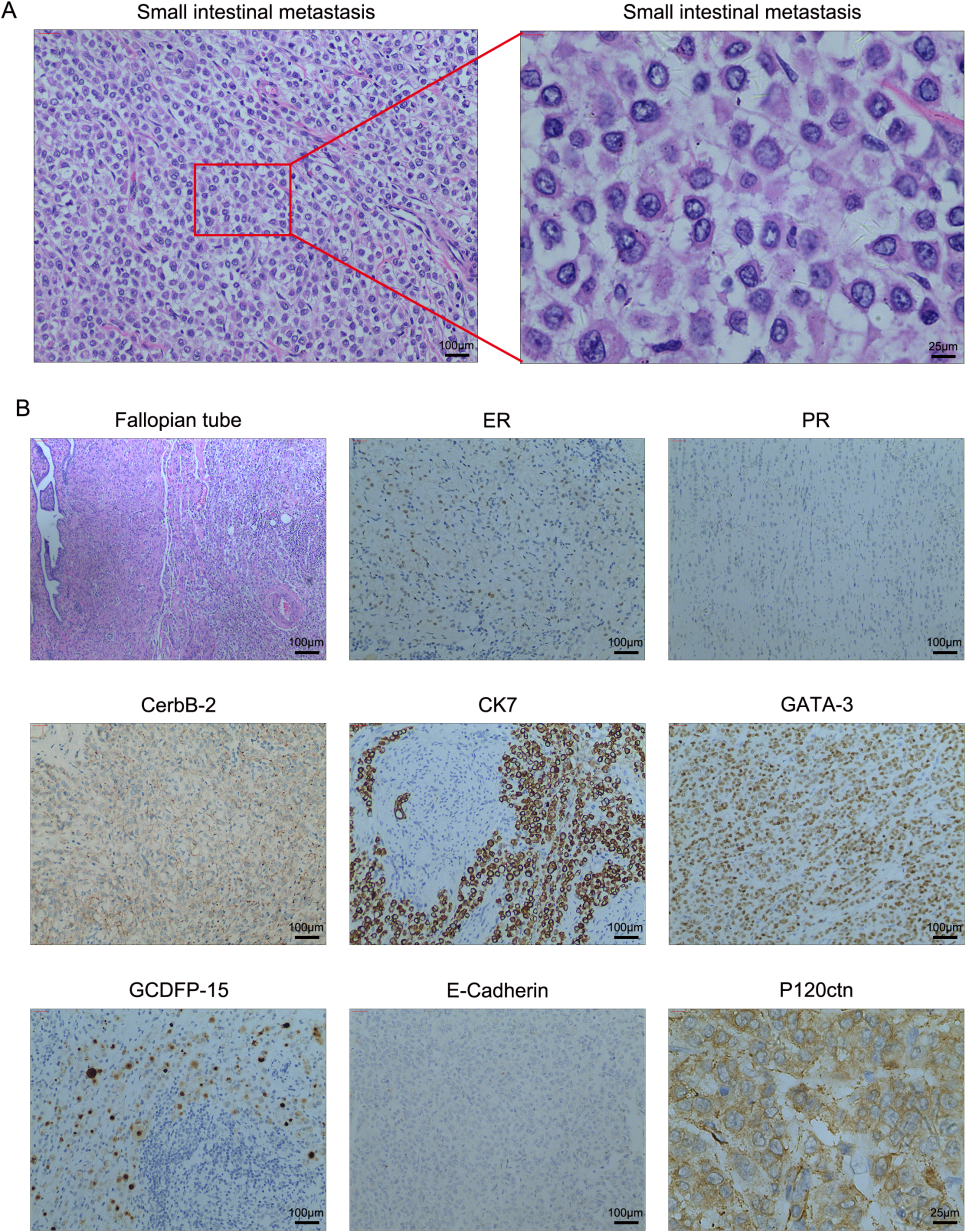
**(A)** High-resolution HE images of the small intestinal metastasis. **(B)** The representative images of immunohistochemistry and H&E staining in the small intestine and right fallopian tube tissues. Magnification, 100×; scale bar, 100μm and magnification, 400×; scale bar, 25μm.

During the surgical procedure, intestinal contracture and stenosis were observed within the abdominal cavity, with subsequent dilation of the terminal ileum. Approximately 200 ml of slightly yellow ascites was noted in the pelvic cavity. Additionally, tumor metastasis was identified in the small intestine, 100 cm distal to the ileocecal region. Both ovaries were found to be atrophic. A partial ileal resection and bilateral oophorectomy were performed. The resected tumor measured 1×1.5 cm and histological examination revealed pleomorphic lobular carcinoma infiltrating all layers of the small intestine. While no cancer metastases were present in the bilateral ovarian tissues, metastatic disease was identified in the right fallopian tube. Periintestinal lymph nodes were negative for metastases, and the intestinal resection margins were tumor-free. Immunohistochemical analysis revealed the following neoplastic cell characteristics: ER (-), PR (-), CerbB-2 (++), Ki-67 (+, 35%), E-Cadherin (-), GATA-3 (+), CK7 (+), P120ctn (+), and GCDFP-15 (+). Fluorescent *in-situ* hybridization (FISH) analysis demonstrated a mean HER-2 signal of 3.3 and a mean CEP17 signal of 2.4, indicating HER-2 negativity. Representative images of HE and immunohistochemical staining in the small intestine are depicted in **Figure 2**. A re-evaluation of abdominal enhanced CT revealed multiple metastases to the bilateral ribs, spine, and pelvic bones, with no significant signs of abdominal metastases. The patient was discharged and received targeted therapy with trastuzumab deruxtecan (T-DXd) 5.4mg/kg for nine cycles. At the 1-year follow-up, the patient remained alive with multiple bone metastases. Future follow-ups are scheduled every 3 months.

## Discussion

Metastasis of breast cancer to the digestive system, particularly the small intestine, is a relatively uncommon occurrence. Due to the rapid turnover and transit time of small intestinal cells, the lack of bacterial degradation activity, the nature of the epithelial lining, and the relatively dilute alkaline environment, the small intestine has been considered a less favorable site for carcinogenesis. Among small intestinal cancers, metastatic tumors account for approximately 10%, with varying clinical presentations (7). Common sources of these metastases include uterine cancer, cervical cancer, colon cancer, lung cancer, breast cancer, and melanoma (7–9). The prevailing hypothesis suggests that small intestinal metastasis from breast cancer or other solid tumors occurs through hematogenous dissemination and micrometastasis from the primary tumor (7, 10). However, the underlying mechanisms of small intestinal metastasis remain incompletely understood. Diagnosis and treatment of small intestinal metastasis depend on the histological characteristics and stage of the primary tumor. Chemotherapy, such as fluoropyrimidine-oxaliplatin combinations, remains the first-line treatment option (11, 12). The presence of small intestinal metastasis is associated with lower tumor-free survival and overall survival rates, even with early detection and targeted resection or chemotherapy (13). A study by Legué et al. (11) reported a median overall survival of 9.3 months in patients receiving palliative chemotherapy, compared to 3.0 months in those who did not. Targeted therapy, such as Bevacizumab, has been explored as a first-line treatment for metastatic small intestinal cancer. However, the addition of Bevacizumab to first-line palliative chemotherapy has shown limited clinical benefit in terms of overall survival (14). Nonetheless, ongoing advancements in targeted therapy offer the potential to improve patient survival and provide more effective treatment options (15, 16).

Small intestinal metastases from breast cancer may exhibit a distinct genetic profile compared to other digestive organs. In contrast to colorectal cancer, several studies have identified alterations in the *HER2* gene in small intestinal metastases (17, 18). In this case, the patient received T-DXd following the metastasis of breast cancer to the small intestine. According to the American Society of Clinical Oncology/College of American Pathologists Clinical Practice Guideline, the patient’s HER2 status was classified as 2+, with negative FISH results, consistent with a diagnosis of low HER2 expression in breast cancer (19). For patients with negative hormone receptors and low HER2 expression, recommended first-line chemotherapy regimens include single-agent sequential chemotherapy or combined chemotherapy, typically involving anthracycline or/and paclitaxel-based treatments. Chemotherapy combined with immune checkpoint inhibitors, such as paclitaxel + pembrolizumab (PD-L1 CPS ≥ 10), can also be considered as first-line therapy (20, 21). However, patients with off-label use of PD-1 antibody therapy should still be carefully selected in clinical practice. Out of an abundance of caution, our hospital cannot yet use medication beyond the prescribed limit, so we did not consider PD-1 antibody therapy as a treatment strategy. Given the patient’s poor tolerance to chemotherapy and fear of the treatment, we initially recommended chemotherapy but ultimately opted for T-DXd as a first-line drug to address HER2-low-expressing triple-negative breast cancer.

As a novel ADC drug coupled with a TOP1 inhibitor, T-DXd is indicated for the treatment of adult patients with unresectable or metastatic HER2-low breast cancer who have received prior anti-HER2 therapies (22). The potent antitumor effects of T-DXd have been demonstrated in several patient-derived xenograft models, including breast and gastric cancers with low HER2 expression as well as tumors with heterogeneous HER2 expression. An open-label phase II study enrolled 267 patients treated with T-DXd (5.4 mg/kg once every 3 weeks), demonstrating its efficacy in HER2-expressing solid tumors across seven tumor types: endometrial, cervical, ovarian, bladder, biliary tract, pancreatic, and others (23). In HER2-low metastatic breast cancer, the ASCENT and DESTINY-Breast04 phase III trials established T-DXd as a strong competitor to sacituzumab govitecan (SG) as a second-line treatment for patients with HER2-low metastatic triple-negative breast cancer (24). Furthermore, the DESTINY-CRC01 trial, a multicenter, open-label, phase II study evaluating patients with HER2-expressing metastatic colorectal cancer, reported that patients receiving T-DXd 6.4 mg/kg every 3 weeks achieved an objective response rate of 45.3%, a disease control rate of 83.0%, a median progression-free survival of 6.9 months, and a median overall survival of 15.5 months, suggesting robust and durable antitumor activity in patients with HER2-positive metastatic colorectal cancer (25). Additionally, T-DXd has shown potential activity in combination with other HER2-targeted regimens and is considered a subsequent-line therapy for HER2-positive, RAS-mutated metastatic colorectal cancer (26). While large clinical studies specifically addressing breast cancer metastasis to the small intestine are currently lacking, current treatment primarily relies on clinical guidelines for metastatic colorectal cancer from breast cancer. Based on the promising clinical trial results of T-DXd in treating HER2-expressing breast and colorectal cancers, T-DXd therapy may be an effective treatment strategy for patients with small intestinal metastasis from breast cancer and our case report may contribute to the clinical experience of treating this condition with T-DXd (23, 27).

Our case report highlights several intriguing pathological phenomena. As presented, GCDFP-15 positivity was observed in both primary breast cancer and metastatic small intestinal tumors. Given the established association of GCDFP-15 positivity with breast origin in metastatic tumors, we concluded that the small intestinal metastasis originated from primary breast cancer (28, 29). However, notable differences were evident between the immunohistochemical profiles of the primary tumor and metastatic lesions. Based on the HE results, we hypothesized that the metastatic tumor might represent pleomorphic lobular carcinoma, which could explain the discordance in hormone and HER2 status. High-resolution HE images were examined to verify this hypothesis. As shown in **Figure 2**, the HE findings in the metastatic tumors support the diagnosis of pleomorphic lobular carcinoma. The pleomorphic nature of breast pleomorphic lobular carcinoma, characterized by atypical cells with diverse nuclei, may account for the discordance in hormone receptor and HER2 status between the primary and metastatic lesions. The heterogeneity of breast cancer tumor cells has led to studies demonstrating changes in hormone receptor and HER2 status in some patients with recurrence or metastasis. Compared to the conversion from negative to positive receptor expression, the transition from receptor positive to negative is more common and often associated with resistance to original treatments and a poor prognosis (30–32). For example, a study by Francis et al. (33) found that 34.2% of ER+, PR+ primary breast cancer patients developed ER-, PR- metastatic tumors, and 21.9% of HER2-negative primary breast cancer patients exhibited HER2+ metastatic tumors. Reassessing receptor status in breast cancer recurrence and metastasis, guiding clinical decisions based on receptor changes, and selecting appropriate targeted drugs or immunotherapy are crucial for adjusting treatment strategies and predicting prognosis.

Research on breast cancer metastasis to the intestinal tract remains limited. We have compiled a summary of relevant reports published over the past 20 years in **Table 1**. Invasive lobular carcinoma (ILC) of the breast is generally considered to have a higher propensity for intestinal metastasis. As reported by McLemore et al. (34), gastrointestinal metastases are more common in patients with invasive lobular carcinoma. While surgical intervention may not significantly prolong overall survival, it can be considered in selected cases. Compared to invasive ductal carcinoma (IDC), invasive lobular carcinoma is more likely to exhibit a loss of E-cadherin, a marker of cell-to-cell adhesion, which may contribute to more diffuse infiltration of affected gastrointestinal organs by primary and metastatic ILC (35). However, approximately 10% of lobular carcinomas may demonstrate an abnormal E-cadherin expression pattern characterized by incomplete, fragmented, or bead-like membranous staining. Cytoplasmic staining may also be diffuse or accompanied by perinuclear punctate staining (36). Additionally, ER+, CK7+, and CK20+ expression in primary breast cancer have been associated with a higher likelihood of intestinal metastasis (37). The immunohistochemical profile of primary breast cancer prior to intestinal metastasis, as reported in the literature review, is summarized in **Table 2**. However, compared to the comprehensive and detailed immunohistochemical results presented in case reports of breast cancer colon metastasis, many important immunohistochemical markers are not mentioned in case reports of small intestinal metastasis.

**Table 1:** Clinical cases of intestinal metastasis from primary breast cancer.

Year (Ref)	Age	Patients (n)	Primary breast cancer	Metastasis site	Abdominal symptoms	Treatment	Outcome
2005 (19)	49	1	ILC	jejunum	Diffuse colic abdominal pain with Intestinal obstruction	Chemotherapy and endocrine therapy	Alive for 6 months after diagnosis
2007 (20)	72	1	ILC	ileum	Intestinal obstruction	Right hemicolectomy	NR
2007 (21)	72	1	ILC	ileum	Acute abdominal pain, bloating, and difficulty defecating	Ileocolic anastomosis	NR
2007 (22)	62	1	SRCC	Duodenum	Duodenal obstruction and acute pancreatitis	Palliative surgery and anthracycline-based chemotherapy followed by docetaxel	Alive for 18 months since first admission
2011 (23)	60	1	ILC	ileum	Abdominal pain and distention	Right hemicolectomy and ileocolic anastomosis and hormonal therapy	NR
2011 (24)	79	1	IDC	NR	acute onset of lower abdominal pain	Partial resection of the small intestine and primary anastomosis	Three months after surgery due to multiple liver metastases
2013 (25)	41	1	ILC	ileum	Recurrent abdominal pain with bloating	Small bowel resection and anastomosis and two cycles of XT chemotherapy and endocrine therapy	NR
2015 (26)	50	1	IDC	Duodenum	Duodenal obstruction	Tumor resection and gastrojejunostomy and Taxotere with Herceptin for 6 cycles	Brain metastases after 3 months of diagnosis and alive 7 months after radiation therapy for brain metastases.
2018 (27)	56	1	ILC	jejunum	Cramp-like intermittent abdominal pain with nausea and vomiting symptoms	Jejunum segmental resection and jejunojejunostomy and endocrine therapy	NR
2018 (28)	56	1	IDC	jejunum and ileum	progressive bellyache and vomiting	Radical excision and Cedaramine CDM301 combined with Exemestane therapy	Alive for 20 months after diagnosis
2020 (29)	64	1	ILC	Ileocecal region	Abdominal pain, nausea, emesis and stool changes	Right-sided hemicolectomy and creation of an end ileostomy	Died on the 25th postoperative day
2021 (30)	60	1	ILC	ileum	Intermittent colicky abdominal pain and distension	Small bowel resection and anastomosis and oncological treatment	Alive for 6 months after diagnosis
2022 (5)	49	1	ILC	ileum	Upper abdominal pain and abdominal distention	Partial resection of the small intestine and six cycles of chemotherapy with paclitaxel liposomes combined with carboplatin	NR
2022 (31)	58	1	MPT	jejunum	Paroxysmal moderate periumbilical pain accompanied by a cessation of exhaust and defecation	Without further treatment	Without further follow-up
2024 (32)	69	1	ILC	ileum	Abdominal pain, vomiting, and abdominal distention	NR	NR
2024 (33)	69	1	ILC	jejunum and ileum	Constipation and vomiting	Jejunoileal resection with end-to-end jejunoileal and side-to-side ileosigmoid anastomoses	Died on the 28th postoperative day
2024 (34)	72	1	MPT	ileum	Intestinal obstruction	Ileocaecal resection and ileocolonic anastomosis	Dead after 14th day of diagnosis

IDC, Invasive ductal carcinoma; ILC, invasive lobular carcinoma; MPT, Malignant phyllodes tumors; SRCC, breast signet-ring cell carcinoma; NR, not reported.

**Table 2:** Immunohistochemistry result of intestinal metastasis from primary breast cancer.

Year (Ref)	Primary breast cancer	Metastasis site	Immunohistochemistry result of intestinal metastasis from primary breast cancer								
			ER	PR	Her-2	Ki67	E-cadherin	CK7	CK20	GATA-3	GCDFP-15
2005 (19)	ILC	jejunum	+	+	+	Low	NR	+	–	NR	NR
2007 (20)	ILC	ileum	+	+	NR	NR	NR	+	NR	NR	NR
2007 (21)	ILC	ileum	NR	NR	NR	NR	NR	NR	NR	NR	NR
2007 (22)	SRCC	Duodenum	–	–	–	NR	–	NR	NR	NR	NR
2011 (23)	ILC	ileum	+	+	–	NR	NR	+	NR	NR	+
2011 (24)	IDC	NR	–	–	–	NR	NR	NR	NR	NR	NR
2013 (25)	ILC	ileum	+	+	–	+5‐10%	NR	+	NR	NR	+
2015 (26)	IDC	Duodenum	+	–	+	NR	NR	+	NR	NR	NR
2018 (27)	ILC	jejunum	+	+	–	NR	NR	NR	NR	NR	NR
2018 (28)	IDC	jejunum and ileum	+	–	–	10%	NR	+	–	NR	+
2020 (29)	ILC	Ileocecal region	+	–	–	NR	NR	+	–	+	NR
2021 (30)	ILC	ileum	+	+	–		NR	NR	NR	+	NR
2022 (5)	ILC	ileum	+	+	+	5%	NR	+	–	+	+
2022 (31)	MPT	jejunum	NR	NR	NR	NR	NR	NR	NR	NR	NR
2024 (32)	ILC	ileum	+	NR	NR	NR	NR	+	NR	NR	NR
2024 (33)	ILC	jejunum and ileum	+	NR	+	NR	NR	NR	NR	NR	NR
2024 (34)	MPT	ileum	NR	NR	NR	NR	NR	NR	NR	NR	NR

IDC, Invasive ductal carcinoma; ILC, Invasive lobular carcinoma; MPT, Malignant phyllodes tumor; SRCC, breast signet-ring cell carcinoma, NR, not reported. ER, Estrogen receptors; PR, Progesterone receptors.

+ indicates positive result in immunohistochemistry.

– indicates negative result in immunohistochemistry.

Given the current understanding of organ metastasis, systemic chemotherapy, and surgical treatment remain the primary options for the management of intestinal metastasis. Targeted therapy, such as trastuzumab, has also been reported in recent case studies (38, 39). We recommend that surgeons consider differential diagnosis and immunohistochemical evaluation of malignancy in patients with any intestinal symptoms and a history of breast cancer, particularly those with a diagnosis of lobular breast cancer, regardless of the duration of the disease-free interval. This approach may facilitate earlier diagnosis and treatment.

## Conclusion

Patients with a history of breast cancer, particularly those diagnosed with lobular breast cancer, should be carefully evaluated for intestinal metastasis if they present with any intestinal symptoms. Re-evaluation of receptor status in breast cancer metastases is crucial for guiding adjustments in therapeutic strategies. For patients with HER2-low expression, a combination of targeted therapy and palliative resection of small intestinal lesions may potentially improve survival. Further research is required to elucidate the optimal combined strategy of chemotherapy, radiotherapy, and biotherapy for the standardized management of small intestinal metastasis from breast cancer.

## Data Availability

The original contributions presented in the study are included in the article/**Supplementary Material**. Further inquiries can be directed to the corresponding author.

## Supplementary material

Supplementary Figure 1 The representative images of **(A)** CT, **(B)** colonoscopy, and **(C)** PET-CT.
