# Supplementary figures and images for: Small intestinal metastasis from primary breast cancer: a case report and review of literature
Source: Front Immunol. 2024 Dec 3;15:1475018. doi: 10.3389/fimmu.2024.1475018 (PMC11653178; doi:10.3389/fimmu.2024.1475018)

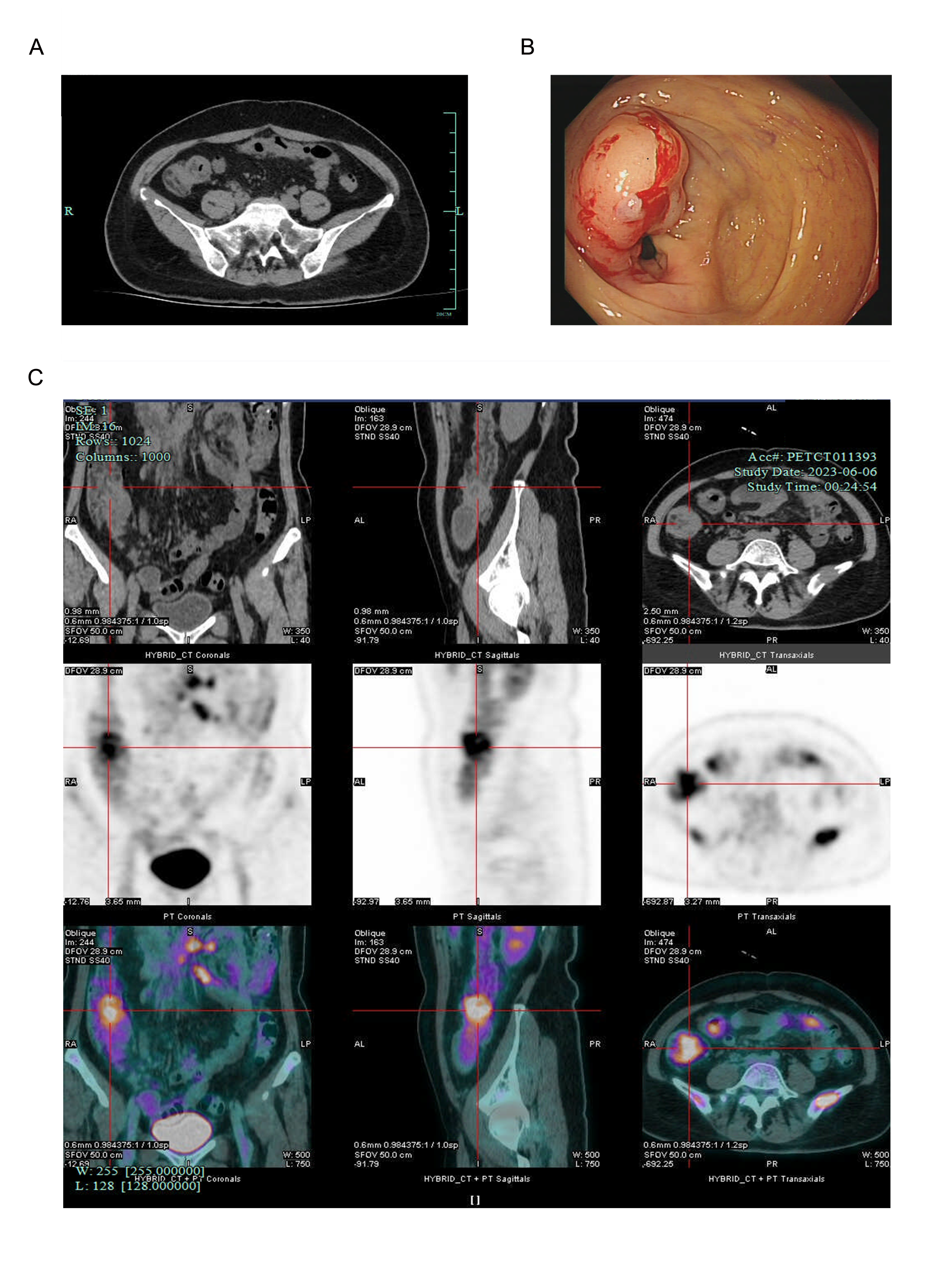

Supplement: Supplementary Figure 1 — The representative images of (A) CT, (B) colonoscopy, and (C) PET-CT. [file Image1.tif]
